# Adolescent cohorts assessing growth, cardiovascular and cognitive outcomes in low and middle-income countries

**DOI:** 10.1371/journal.pone.0190443

**Published:** 2018-01-16

**Authors:** Joseph L. Ward, Katherine Harrison, Russell M. Viner, Anthony Costello, Michelle Heys

**Affiliations:** 1 UCL Institute of Child Health, University College London, London, United Kingdom; 2 University College London Hospitals NHS Foundation Trust, London, United Kingdom; 3 UCL Institute for Global Health, University College London, London, United Kingdom; 4 Department of Maternal, Newborn, Child and Adolescent Health (MCA) World Health Organization, Geneva, Switzerland; London School of Hygiene and Tropical Medicine, UNITED KINGDOM

## Abstract

**Introduction:**

Life-course studies are needed to explore how exposures during adolescence, particularly puberty, contribute to later cardiovascular risk and cognitive health in low and middle-income countries (LMIC), where 90% of the world’s young people live. The extent of any existing cohorts investigating these outcomes in LMIC has not previously been described.

**Methods:**

We performed a systematic literature review to identify population cohort studies of adolescents in LMIC that assessed anthropometry *and* any of cardiovascular risk (blood pressure, physical activity, plasma glucose/lipid profile and substance misuse), puberty (age at menarche, Tanner staging, or other form of pubertal staging) or cognitive outcomes. Studies that recruited participants on the basis of a pre-existing condition or involved less than 500 young people were excluded.

**Findings:**

1829 studies were identified, and 24 cohorts fulfilled inclusion criteria based in Asia (10), Africa (6) and South / Central America (8). 14 (58%) of cohorts identified were based in one of four countries; India, Brazil, Vietnam or Ethiopia. Only 2 cohorts included a comprehensive cardiovascular assessment, tanner pubertal staging, *and* cognitive outcomes.

**Conclusion:**

Improved utilisation of existing datasets and additional cohort studies of adolescents in LMIC that collect contemporaneous measures of growth, cognition, cardiovascular risk and pubertal development are needed to better understand how this period of the life course influences future non-communicable disease morbidity and cognitive outcomes.

## Introduction

Almost a quarter of the global population is aged between 10–24 years, 90% of whom live in developing countries[1]. The burden of mortality and morbidity within this population is more significant than previously recognized, and very largely preventable[2]. The health of adolescents and young people are intrinsically linked to those of the next generation, and it is during this stage of the life course where we have the greatest opportunity to influence the future burden of non-communicable disease, and the adoption of risk behaviours which may determine other health outcomes in adulthood. It is during adolescence where unhealthy behaviours associated with cardiovascular disease such as smoking and alcohol use are initiated and established[3], where mental health problems first arise, and where risks associated with pregnancy and birth are greatest. Of the 10 leading causes of disability adjusted life years (DALY) identified in the global burden of disease study, 6 are determined during this period of the life course[4]. There is now growing international recognition of the importance of investing in adolescent health, particularly in low and middle-income countries (LMICs). To do so will bring a triple dividend: benefits now, for future adult health, and for the next generation[5].

Despite this importance to global development, data collection and studies of adolescent health are very limited outside high-income countries. As adolescent health outcomes were largely absent from the Millennium Development Goal agenda, and this period of the life course has been perceived as one of relative health, the importance of collecting data within this age group, particularly within resource poor settings, has previously been overlooked. Young people are often not included in data collection systems that frequently do not disaggregate data by age across the early life course[6]. Further, indicators describing the social contexts in which adolescents live, study and work, known to be powerful determinants of morbidity and mortality in this age group, often do not feature in global data systems. There is an acknowledged need to develop capacity in national statistical systems in LMIC and expand adolescent participation in research to better understand this crucial stage of the life course[6].

Collecting data through life-course studies of exposures during puberty is an essential part of this. Puberty is a time of immense physiological and psychological change where future health and social trajectories are set in motion[7]. The timing of onset of puberty has been shown to influence multiple health outcomes[7], and the interaction between how growth and pubertal changes impact cardiovascular risk and cognitive outcomes differently is of particular interest.

Adult cognitive outcomes, particularly in language, memory and executive function, are heavily linked to exposures during childhood and adolescence[8] and these may be more important than those during the prenatal period or later in adulthood[9]. Pubertal development and growth during adolescence are influential in this; the timing of puberty impacts directly on grey and white matter development[10] and earlier puberty in women may be protective for adulthood cognitive function[8, 11]. However, the benefit to brain development of early puberty may come at the cost of worse cardiovascular outcomes later in life. This could represent an example of the theory within evolutionary biology of “life-history trade-offs,” where an organism sacrifices long-term life expectancy to prioritise short term survival and greater likelihood of reproductive success[12]. Early menarche is associated with increased body mass index (BMI) in adolescence and adulthood, after adjusting for childhood BMI[13] and early puberty has also been associated with increased BMI and blood pressure in males, independent of pre-pubertal weight, birth-weight or social class[14]. A recent systematic review also found early puberty to be associated with cardiovascular mortality, hypertension, metabolic syndrome, abnormal glycaemia and obesity[15]. Certain patterns of growth during adolescence, childhood and infancy are also associated with increased cardiovascular risk[16]. For example, greater sitting height, primarily determined during adolescence, was associated with increased risk of diabetes and dyslipidaemia in one Chinese cohort[17]. Exploring these relationships will require longitudinal studies of adolescence collecting data on these outcomes simultaneously.

Much of the evidence for these associations comes from studies in high–income settings, and extrapolating their findings to LMICs is problematic for many reasons. Firstly, the prevalence of exposures and outcomes pertinent to adolescent health, growth, puberty and future cardiovascular and cognitive outcomes, such as poor nutritional status and tobacco, alcohol and substance use, varies by income group[6]. The nature of exposures may also differ, for example a third of tobacco intake in South Asia is smokeless[18]. Whereas physical activity in LMICs is primarily due to commuting, manual labour, or domestic work, in high-income countries this is related to leisure activities. Further, the confounding structure of exposures and outcomes will vary; the protective role of breastfeeding on childhood adiposity shown in developed countries have not been replicated in non-European settings, suggesting socioeconomic status was confounding this association[19]. Socio-economic inequalities in cardiovascular disease are also dependent on level of economic development[20], with childhood obesity being common amongst low socioeconomic groups in wealthy countries, and in high socioeconomic groups in the developing world[21]. Body composition may also vary for a given body size, and have differing influence on non-communicable risk. For example, low birth weight infants from LMICs may have a “thin-fat” phenotypic body composition that can increase risk of type 2 diabetes[22].

In order to assess the need for further longitudinal studies investigating these outcomes outside high-income countries, we performed a systematic literature review to identify cohort studies of adolescents in LMICs that include assessments of growth, cardiovascular risk, pubertal development and cognition. We hypothesised there would be limited availability of cohort studies assessing these variables in LMICs.

## Methods

### Search strategy

We performed a systematic literature search on 24^th^ Feb 2016 of MEDLINE. Keywords (MeSH and text words) used to describe the study population were: adolescent, adolescence, young person, teenager, youth and children (see Appendix). Keywords used to identify relevant study design were: birth cohort, panel study, longitudinal study and cohort study. Keywords used to identify studies in relevant settings were: low and middle income country, low income country, middle income country, developing country, and the individual country names for all those defined as low or middle income using the World Bank lending classification of Gross National Income (GNI) per capita of less than $12735 in 2015[23]. Reference lists of selected papers were then searched for any further relevant studies.

### Data extraction

We used the online literature review tool www.covidence.org to extract the data. Inclusion criteria are listed in Table 1. An article was eligible if it referred to a longitudinal study design, was set in a LMIC, included participants aged between 10 and 19 years and assessed anthropometry (objectively recorded) *and* any of cardiovascular risk (any one of: blood pressure, physical inactivity, plasma glucose/lipid profile or substance misuse), puberty (age at menarche, Tanner staging, or other form of pubertal staging) or cognitive outcomes. Cohorts were included regardless of recruitment method or population representativeness. Cohorts with less than 500 participants were excluded (as they are unlikely to have sufficient power to examine associations between growth, puberty, cardiovascular risk and cognitive outcomes), as were cohorts that recruited participants on the basis of a pre-existing condition. Multiple papers referring to the same cohort were also excluded. Non-English language papers were not excluded, (although none were identified).

**Table 1:** Inclusion criteria.

**Primary Inclusion Criteria**	**Exclusion Criteria**
Birth cohort / longitudinal cohort	Less than 500 participants
• Or cross-sectional study of longitudinal population	Participants recruited on basis of pre-existing medical condition
• Or review of longitudinal population	
Low or Middle Income Country	
Participants aged between 10 and 19 years	
**Secondary Inclusion Criteria**	
Anthropometry *and* cardiovascular, pubertal or cognitive outcomes	

Details of cohort sample size, retention rate, birth year, data availability, and whether data were collected during mid-childhood and the perinatal period in addition to adolescence (ages 10–19) were recorded. Papers summarising the cohort profile were identified where possible. Each selected cohort was critiqued and rated good, poor or fair by two authors (JLW and KH) using the Quality Assessment Tool for Observational Cohort and Cross-Sectional Studies provided by the U.S Department of Health and Human Services[24].

## Results

**Fig 1:**
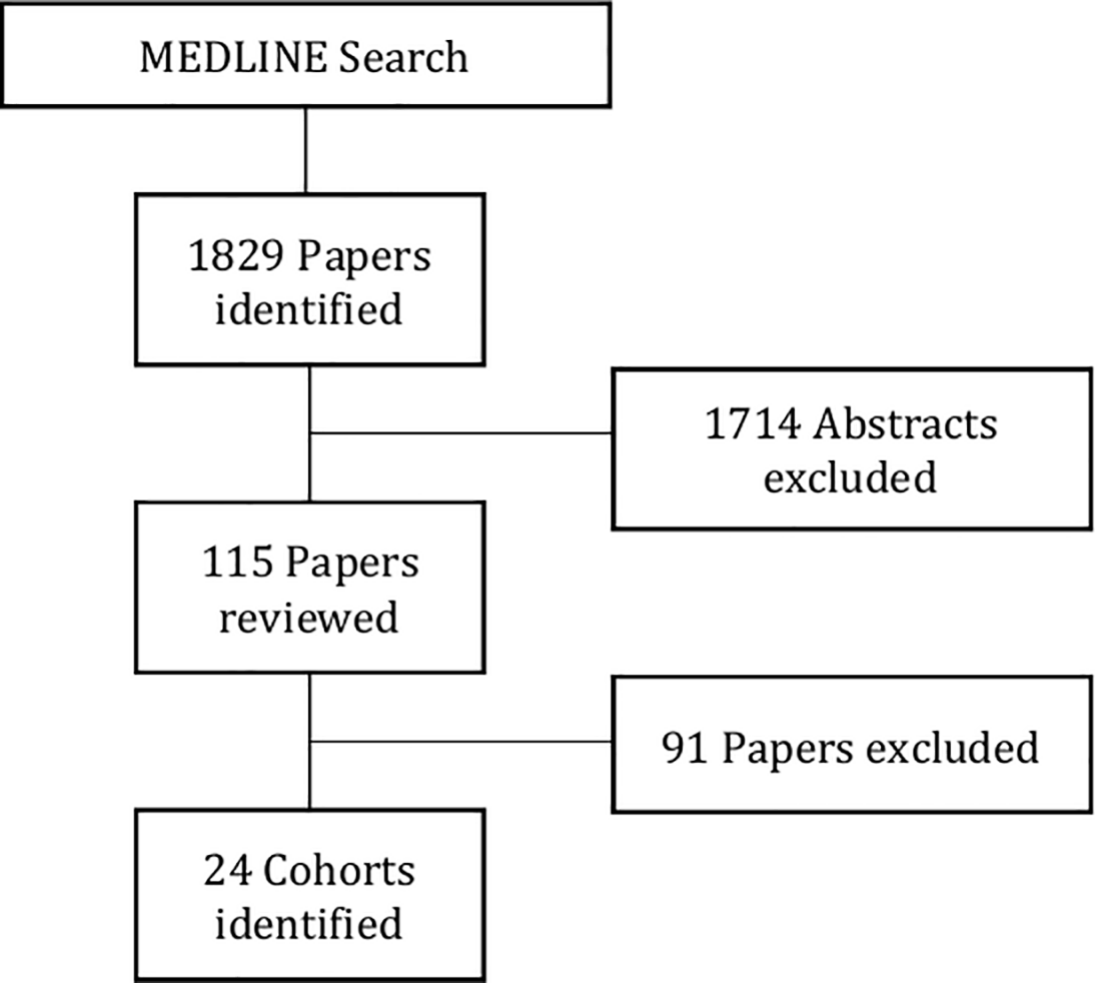
Literature review search strategy.

We reviewed abstracts from 1829 papers, of which 1714 did not fulfil the inclusion criteria, or referred to cohorts already selected. Reviewing the full texts of the remaining 115 papers excluded a further 91 papers (Fig 1.)

We identified 24 adolescent cohorts from 12 countries across Asia (n = 10), Africa (n = 6) and South/ Central America (n = 8) (Tables 2,3 and 4). 21 cohorts were rated as “good” using the Quality Assessment Tool for Observational Cohort and Cross-Sectional Studies (see Appendix), with 1 cohort rated “fair” (the INCAP Longitudinal Study[25]), 1 as “good/fair” (The Tsimane Amazonian Panel Study (TAPS)[26]) one as “fair/poor” (the Chinese Metabolic Syndrome Twin Cohort Study[27]). Tables 2, 3 and 4 show a summary of each cohort identified, by geographical region. 10 cohorts included variables to assess all the variables of interest (growth, puberty, cognition and cardiovascular risk): (The Mysore Parthenon Birth Cohort,[28] Cebu Longitudinal Health and Nutrition Survey[29], Pelotas (1993) Cohort[30, 31], the younger child Young Lives[32] cohorts in Vietnam, Ethiopia, Peru and India, the Birth to Twenty Cohort[33], Cape Area Panel Study(CAPS)[34] and the 1986 Jamaica Birth Cohort[35]). Of these, 2 included a broad cardiovascular risk assessment (BP, glucose and lipid profile, physical activity and anthropometry), Tanner pubertal staging, and a range of variables to assess cognition (the Birth to Twenty Cohort[33] and the Mysore Parthenon Birth Cohort[28]).

**Table 2:** Characteristics of and outcomes assessed in adolescent cohorts in low and middle income countries in Asia.

Name	Country	Birth Years	Sample Size	Retention^i^	Perinatal period	Mid Childhood	Cognition	Puberty	Cardiovascular Risk	Data availability
South Delhi Cohort[36]	India	1969/72	4092	2672 65%	**✓**	**✓**	IQ testing^**ii**^	x	BP, glucose, & lipid profile, substance use, anthropometry	Not freely available, contact authors
Andhra Pradesh Children and Parents Study[37]	India	1987/90	2601	1446 56%	x	x	x	Tanner staging (Self-assessed)	BP, glucose & lipid profile, physical activity, DEXA scan, anthropometry	Not freely available, contact authors
Mysore Parthenon Birth Cohort[28]	India	1997/98	663	545 82%	**✓**	**✓**	Kaufman Assessment Battery for Children, Kohs block-design test, Coding-Wechsler Intelligence Scale for Children	Tanner staging (Physician-rated)	BP, glucose & lipid profile, physical activity, anthropometry	Not freely available, contact authors
Young Lives[32] (Younger Child)	India	2000/01	2011	1915 95%	x	**✓**	Peabody Picture Vocabulary Test (PPVT) and the Cognitive Developmental Assessment (CDA), maths and literacy assessment	Pubertal status (Self-assessed), age at menarche	Physical activity, substance misuse, anthropometry	Available from http://younglives.qeh.ox.ac.uk
Young Lives[32] (Older Child)	India	1994/95	1008	952 94%	x	**✓**	Peabody Picture Vocabulary Test (PPVT) and the Cognitive Developmental Assessment (CDA), maths and literacy assessment	x	Physical activity, substance misuse, anthropometry	Available from http://younglives.qeh.ox.ac.uk
Young Lives[32] (Younger Child)	Vietnam	2000/01	2002	1932 97%	x	**✓**	Peabody Picture Vocabulary Test (PPVT) and the Cognitive Developmental Assessment (CDA), maths and literacy assessment	Pubertal status (Self-assessed), age at menarche	Physical activity, substance misuse, anthropometry	Available from http://younglives.qeh.ox.ac.uk
Young Lives[32] (Older Child)	Vietnam	1994/95	1000	887 89%	x	**✓**	Peabody Picture Vocabulary Test (PPVT) and the Cognitive Developmental Assessment (CDA), maths and literacy assessment	x	Physical activity, substance misuse, anthropometry	Available from http://younglives.qeh.ox.ac.uk
Ho Chi Minh City Youth Cohort[38]	Vietnam	2004	759	585 77%	x	x	x	Pubertal status (Self-assessed), age at menarche	BP, glucose & lipid profile, physical activity, anthropometry	Available, contact nguyenhoang_doantrang@yahoo.com
Cebu Longitudinal Health and Nutrition Survey[29]	Philippines	1983/84	3327	1817 59%	**✓**	**✓**	IQ testing	Pubertal status (Self-assessed)	BP, glucose & lipid profile, physical activity, anthropometry	Available from http://www.cpc.unc.edu/projects/cebu
Chinese Metabolic Syndrome Twin Cohort Study[27]	China	1998–2000	953	953 (100%)	x	**✓**	x	Tanner staging (Physician-rated)	Physical activity, lipid profile, fat composition using DEXA scan, anthropometry	Not freely available, contact authors

^i^retention during adolescence (10–19)

^ii^6-8 year olds only

**Table 3:** Characteristics of and outcomes assessed in adolescent cohorts in low and middle income countries in South America and Caribbean.

Name	Country	Birth Years	Sample Size	Retention^i^	Perinatal period	Mid Childhood	Cognition	Puberty	Cardiovascular Risk	Data availability
Pelotas Birth Cohort[39]	Brazil	1982	5914	1597 (27%)^ii^	**✓**	**✓**	x	x	Substance misuse, blood pressure, physical activity, anthropometry	Not freely available, contact authors
Pelotas Birth Cohort[30, 31]	Brazil	1993	5265	4106 (78%)	**✓**	**✓**	IQ testing	Age at menarche	BP, glucose & lipid profile, physical activity, substance misuse, anthropometry	Not freely available, contact authors
Riberao Preto Birth Cohort[40]	Brazil	1978/79	6827	2063 (30%)	**✓**	**✓**	x	x	BP, glucose & lipid profile, physical activity substance use, anthropometry	Not freely available, contact authors
Young Lives[32] (Younger Child)	Peru	2000/01	2052	1902 (92%)	x	**✓**	Peabody Picture Vocabulary Test (PPVT) and the Cognitive Developmental Assessment (CDA), maths and literacy assessment	Pubertal status (Self-assessed), age at menarche	Physical activity, substance misuse, anthropometry	Available from http://younglives.qeh.ox.ac.uk
Young Lives[32] (Older Child)	Peru	1994/95	714	635 (89%)	x	**✓**	Peabody Picture Vocabulary Test (PPVT) and the Cognitive Developmental Assessment (CDA), maths and literacy assessment	x	Physical activity, substance misuse, anthropometry	Available from http://younglives.qeh.ox.ac.uk
1986 Jamaica Birth Cohort[35]	Jamaica	1986	10054	2371^iii^ (24%)	**✓**	x	Raven’s Progressive Matrices score, WRAT Spelling, Reading an Arithmetic score, Peabody Picture Vocabulary score	Pubertal status^iv^	BP, physical activity, glucose & lipid profile, substance misuse, anthropometry	Available, contact affette.mccawbinns@uwimona.edu.jm
The Tsimane Amazonian Panel Study (TAPS)[26]	Bolivia	1986 /2000	1453^v^	521^v^ (35.9%)	x	**✓**	x	x	BP, substance misuse, anthropometry	Available from http://alanfschultz.com
INCAP Longitudinal Study[25]	Guatemala	1969/77	2169^vi^	1574 (72.6%)	**✓**	**✓**	Ravens Standard Progressive Matrices, tests of functional performance and information processing	x	BP, physical activity, anthropometry	Not freely available, contact authors

^i^retention during adolescence (10–19)

^ii^79% of male cohort also interviewed aged 18 (n = 3037)

^iii^Birth cohort participants who were attempted to be contacted during adolescence

^iv^puberty assessment method (self-assessed/physician-assessed) unknown

^v^includes entire sample, adults and children. Sample under 16 = 820

^vi^ includes participants with only cross-sectional anthropological data.

**Table 4:** Characteristics of and outcomes assessed in adolescent cohorts in low and middle income countries in Africa.

Name	Country	Birth Years	Sample Size	Retention^i^	Perinatal period	Mid Childhood	Cognition	Puberty	Cardiovascular Risk	Data availability
Jimma Longitudinal Family Survey of Youth[41–43]	Ethiopia	1992–1996	2084	1052 (50.5%)	x	x	x	Age at menarche	x	Not freely available, contact authors
Young Lives[32] (Younger Child)	Ethiopia	2000/01	1999	1875 (94%)	x	**✓**	Peabody Picture Vocabulary Test (PPVT) and the Cognitive Developmental Assessment (CDA), maths and literacy assessment	Pubertal status (Self-assessed), age at menarche	Physical activity, substance misuse, anthropometry	Available from http://younglives.qeh.ox.ac.uk
Young Lives[32] (Older Child)	Ethiopia	1994/95	1000	908 (91%)	x	**✓**	Peabody Picture Vocabulary Test (PPVT) and the Cognitive Developmental Assessment (CDA), maths and literacy assessment	x	Physical activity, substance misuse, anthropometry	Available from http://younglives.qeh.ox.ac.uk
Kagera Health and Development Survey[44]	Tanzania	1975/ 1994	4110^ii^	2877^iii^ (70%) 2926^iv^ (72%)	**✓**	**✓**	x	x	Physical activity, substance misuse, anthropometry	Available from http://econ.worldbank.org
Birth to Twenty[33]	South Africa	1990	3273	2100 (67%)	**✓**	**✓**	Bayley Scales of Infant Development; Griffiths Scales of Mental Development; speech, language and hearing development; Denver Developmental Screening Questionnaire; Ravens Coloured Progressive Matrices	Age at menarche, Tanner pubertal staging (Self- assessed)	BP, glucose & lipid profile, physical activity, substance misuse, DEXA scan, anthropometry	Not freely available, contact authors
Cape Area Panel Study (CAPS)[34]	South Africa	1980–1988	4752	2915 (61%)	x	x	literacy and numeracy assessment	Pubertal status (Self-assessed) age at menarche	BP, substance misuse, anthropometry	Available http://www.caps.uct.ac.za/

^i^retention during adolescence (10–19)

^ii^participants aged <20 in original baseline survey 1991/1994

^iii^followed up in 2004

^iv^followed up in 2010

## Discussion

This systematic literature review identified 24 adolescent cohorts set in LMICs that include data on growth and pubertal timing and cognitive or cardiovascular outcomes. Although these cohorts cover 12 different LMICs, 58% were based in one of only 4 countries; India, Brazil, Vietnam and Ethiopia, and 8 (33%) were from one cross-national study (Young Lives[32]). Aside from these cohorts, we found very limited life-course data available in LMICs that include these indicators of adolescent and future adult health.

We identified ten cohorts which included variables to assess all the outcomes of interest, but only five of these included thorough cardiovascular risk. Of the two which also included Tanner pubertal staging (the Birth to Twenty Cohort[33] and the Mysore Parthenon Birth Cohort[28]), only the Birth to Twenty Cohort[33] has collected data beyond early adolescence.

The cohorts we identified were mostly of high quality, and 22 were rated “good” using the Assessment Tool for Observational Cohort and Cross-Sectional Studies. They varied in size, scope, composition and attrition rates, and were based in countries that reflect a wide range of political, economic, cultural, and social contexts. Although this heterogeneity provides a rich source of diverse data on adolescent health outcomes in these settings, this will limit generalizability. For example, the two South African cohorts identified followed children and young people growing up in a period of unique social and political change in the aftermath of *Apartheid*, limiting the applicability of their findings to other settings. Further, many were not nationally representative. Adolescents from higher socioeconomic groups were under-represented in Young Lives[32], Birth to Twenty[33], CAPS[34], and the 1986 Jamaica Birth Cohort[35], and were more likely to be lost to follow up in the Cebu Longitudinal Health and Nutrition Survey[29] and the Mysore Parthenon Birth Cohort[28]. As discussed, the prevalence and confounding structure of exposures pertinent to adolescent growth and puberty, and subsequent cardiovascular and cognitive risk, varies by socioeconomic group, and so generalising findings from these cohorts may be problematic.

### Comparison with the literature

Our study demonstrates the pressing need for large scale longitudinal cohort studies in LMICs with long follow-up periods, as recently highlighted by Victora and Barros[45]. Their literature search of birth cohorts identified 6500 studies, but of the 20 countries with the largest number of articles, only India and Brazil were LMICs. Where cohort studies are established in these settings, they are often on a small scale with short follow up periods. A systematic review by Campbell et al[46] of birth cohorts in Sub-Saharan Africa identified 28 separate cohorts, but the majority only followed up for less than 2 years. Many were subject to high non-enrolment and attrition rates, and complicated by high rates of migration. Our findings also highlight that non-communicable disease outcomes are poorly represented in longitudinal studies involving adolescents in LMICs, similar to other reviews of cohorts outside high-income countries. A systematic review of birth cohort studies in South East Asia and Eastern Mediterranean regions found only 12 of 83 studies identified focused on non-communicable disease outcomes[47], despite the epidemiological transition shifting the burden of disease away from communicable causes in many of these countries[47].

In addition to establishing new longitudinal studies in LMIC, exploiting existing data sources should also be a priority. The challenges of sharing data sources are well described[48], but initiatives such as The Healthy Birth, Growth, and Development–Knowledge Integration (HBGDki) project[49], which has pooled data from 420 survey studies in 50 countries with the goal of improving interventions for faltering growth and neurocognitive deficits in childhood, demonstrate its potential. The Consortium of Health-Oriented Research in transitional Societies (COHORTS)[50] is a further example of the benefits of collaboration between established study groups. A wider formal network of adolescent cohorts may add insights into the unique role of this period of the life course on future non-communicable disease risk.

### Strengths and limitations

We performed the first systematic literature search identifying longitudinal studies of adolescents in LMIC. We used well-defined inclusion and exclusion criteria to target studies of interest, and reviewed the reference lists of included studies to identify further cohorts. Where possible we utilised published “Cohort Profile” papers, but also accessed the original questionnaires used in the studies where necessary to establish which outcomes were included. There were a number of limitations to our study however. Studies that were initially cross sectional but then followed up at a later date may have been missed. Variables included in the cohorts we identified often change between waves of follow-up, and we may not have described the most recent summary of measurements taken. Although we critiqued the cohorts using the US. Department for Health and Human Services Quality Assessment Tool[24] in order to guide future research using the data sources we identified, multiple other methods are available and may have been more appropriate[51]. We also limited our search to MEDLINE and did not utilise other search engines, which may have biased our results or led us to miss relevant studies. However, as we were trying to identify large well-established cohorts of 500 participants or more, which are referenced in multiple studies, it is unlikely this would have yielded any additional cohorts.

## Conclusion

Improving adolescent health is integral to future adult and newborn outcomes, and yet young people in LMIC continue to be under-represented in research studies[6]. The majority of cohort data identified in this study are freely available or accessible on request, and these sources should be better utilised by researchers in adolescent global health. However, our study demonstrates that additional cohort studies of adolescents in LMIC are needed to establish how exposures during this stage of the life course influence future cognitive outcomes and non-communicable disease morbidity. This should be a global development priority.

## Supporting information

S1 Table Keywords used in the literature search strategy. (PDF)

S2 Table Quality assessment tool used for each cohort included in the study (the quality assessment tool for observational cohort and cross-sectional studies provided by the US department of health and Human Services). (PDF)

S3 Table The PRISMA 2009 checklist for this study. (PDF)

S4 Table Full list of studies identified by the literature search. (XLSX)
