# Supplementary material for: Adolescent cohorts assessing growth, cardiovascular and cognitive outcomes in low and middle-income countries
Source: PLoS One. 2018 Jan 16;13(1):e0190443. doi: 10.1371/journal.pone.0190443 (PMC5770018; doi:10.1371/journal.pone.0190443)
Supplement: S1 Table — (PDF) [file pone.0190443.s001.pdf]

S1 Table: Keywords used in the literature search strategy

**adolescent**
adolescent/or<br>adolescence/or<br>young person/or<br>young people/or<br>teenager/or<br>youth/or<br>children/or
**AND**
**cohort or longitudinal studies**
birth cohort/or<br>panel study/or<br>longitudinal study
**AND**
**low and middle income countries**
low income country\ or<br>low income countries\ or<br>middle income countries\ or<br>middle income countries\ or<br>developing countries\ or<br>developing world\ or<br>afghanistan\ or benin\ or burkina faso\ or burundi\ or cambodia\ or central african republic\ or chad\ or comoros\ or congo, democratic republic\ or congo, democratic republic of the\ or eritrea\ or ethiopia\ or gambia, west africa\ or gambia\ or guinea\ or guinea bissau\ or haiti\ or korea, democratic people s republic of\ or north korea\ or liberia\ or madagascar\ or malawi\ or mali\ or mozambique\ or nepal\ or niger\ or rwanda\ or burundi\ or sierra leone\ or somalia\ or south sudan\ or tanzania\ or togo\ or uganda\ or zimbabwe\ or armenia\ or bangladesh\ or bhutan\ or bolivia\ or cabo verde\ or cameroon\ or congo, republic\ or congo, republic of\ or congo\ or côte d'ivoire \ or ivory coast\ or djibouti\ or egypt\ or el salvador\ or georgia\ or ghana\ or guatemala\ or guyana\ or honduras\ or india\ or indonesia\ or kenya\ or kiribati\ or kosovo\ or kyrgyz\ or kyrgyz republic\ or kyrgyzstan\ or lao pdr\ or lao\ or laos\ or lesotho\ or mauritania\ or micronesia\ or micronesia, federated states of\ or moldova\ or morocco\ or myanmar\ or burma\ or nicaragua\ or nigeria\ or pakistan\ or papua new guinea\ or philippines\ or samoa\ or são tomé and príncipe\ or senegal\ or solomon islands\ or sri lanka\ or sudan\ or swaziland\ or syria\ or tajikistan\ or timor leste\ or east timor\ or ukraine\ or uzbekistan\ or vanuatu\ or vietnam\ or (west bank and gaza\ or palestine\ or yemen\ or zambia\ or albania\ or algeria\ or american samoa\ or samoa\ or angola\ or azerbaijan\ or belarus\ or belize\ or (bosnia and herzegovina\ or botswana\ or brazil\ or bulgaria\ or china\ or colombia\ or costa rica\ or cuba\ or dominica\ or dominican republic\ or ecuador\ or fiji\ or gabon\ or grenada\ or iran\ or iraq\ or jamaica\ or jordan\ or kazakhstan\ or lebanon\ or libya\ or macedonia\ or malaysia\ or maldives\ or marshall islands\ or mauritius\ or mexico\ or mongolia\ or montenegro\ or namibia\ or palau\ or panama\ or paraguay\ or peru\ or romania\ or serbia\ or south africa\ or st lucia\ or (st vincent and the grenadines\ or suriname\ or thailand\ or tonga\ or tunisia\ or turkey\ or turkmenistan\ or tuvalu
