# Supplementary material for: Adolescent cohorts assessing growth, cardiovascular and cognitive outcomes in low and middle-income countries
Source: PLoS One. 2018 Jan 16;13(1):e0190443. doi: 10.1371/journal.pone.0190443 (PMC5770018; doi:10.1371/journal.pone.0190443)
Supplement: S2 Table — (PDF) [file pone.0190443.s002.pdf]

S2 Table: Quality assessment tool used for each cohort included in the study
(the quality assessment tool for observational cohort and cross-sectional studies provided by the US department of health and Human Services).

South Delhi Cohort[51]		
Criteria	JW	KH
(CD, NR, NA)*		
1. Was the research question or objective in this paper clearly stated?	Yes	Yes
2. Was the study population clearly specified and defined?	Yes	Yes
3. Was the participation rate of eligible persons at least 50%?	Yes	Yes
4. Were all the subjects selected or recruited from the same or similar populations (including the same time period)? Were inclusion and exclusion criteria for being in the study prespecified and applied uniformly to all participa	Yes	Yes
5. Was a sample size justification, power description, or variance and effect estimates provided?	No	No
6. For the analyses in this paper, were the exposure(s) of interest measured prior to the outcome(s) being measured?	Yes	Yes
7. Was the timeframe sufficient so that one could reasonably expect to see an association between exposure and outcome if it existed?	Yes	Yes
8. For exposures that can vary in amount or level, did the study examine different levels of the exposure as related to the outcome (e.g., categories of exposure, or exposure measured as continuous variable)?	n/a	Yes
9. Were the exposure measures (independent variables) clearly defined, valid, reliable, and implemented consistently across all study participants?	Yes	Yes
10. Was the exposure(s) assessed more than once over time?	Yes	Yes
11. Were the outcome measures (dependent variables) clearly defined, valid, reliable, and implemented consistently across all study participants?	Yes	Yes
12. Were the outcome assessors blinded to the exposure status of participants?	cd	CD
13. Was loss to follow-up after baseline 20% or less?	No	No
14. Were key potential confounding variables measured and adjusted statistically for their impact on the relationship between exposure(s) and outcome(s)?	Yes	Yes
Quality Rating (Good, Fair, or Poor) (see guidance)	Good	Good
Rater #1 initials:	JW	
Rater #2 initials:		KH
Additional Comments (If POOR, please state why):		
Paper		
Antonisamy B, Raghupathy P, Christopher S, et al. Cohort profile: the 1969-73 Vellore birth cohort study in South India. Int J Epidemiol 2009; **38**(3): 663-9.		
Data Access: Not freely available - collaboration welcomed		

*CD - Cannot determine
*NR - Not reported
*N/A - Not applicable

Andhra Pradesh Children and Parents Study[52]		
Criteria	JW	KH
(CD, NR, NA)*		
1. Was the research question or objective in this paper clearly stated?	Yes	Yes
2. Was the study population clearly specified and defined?	Yes	Yes
3. Was the participation rate of eligible persons at least 50%?	Yes	Yes
4. Were all the subjects selected or recruited from the same or similar populations (including the same time period)? Were inclusion and exclusion criteria for being in the study prespecified and applied uniformly to all participants?	Yes	Yes
5. Was a sample size justification, power description, or variance and effect estimates provided?	cd	CD
6. For the analyses in this paper, were the exposure(s) of interest measured prior to the outcome(s) being measured?	Yes	Yes
7. Was the timeframe sufficient so that one could reasonably expect to see an association between exposure and outcome if it existed?	Yes	Yes
8. For exposures that can vary in amount or level, did the study examine different levels of the exposure as related to the outcome (e.g., categories of exposure, or exposure measured as continuous variable)?	n/a	Yes
9. Were the exposure measures (independent variables) clearly defined, valid, reliable, and implemented consistently across all study participants?	Yes	Yes
10. Was the exposure(s) assessed more than once over time?	Yes	Yes
11. Were the outcome measures (dependent variables) clearly defined, valid, reliable, and implemented consistently across all study participants?	Yes	Yes
12. Were the outcome assessors blinded to the exposure status of participants?	cd	CD
13. Was loss to follow-up after baseline 20% or less?	No	No
14. Were key potential confounding variables measured and adjusted statistically for their impact on the relationship between exposure(s) and outcome(s)?	Yes	Yes
Quality Rating (Good, Fair, or Poor) (see guidance)	Good	Good
Rater #1 initials:	JW	
Rater #2 initials:		KH
Additional Comments (If POOR, please state why):		
Paper		
Kinra S, Krishna KR, Kuper H, et al. Cohort profile: Andhra Pradesh children and parents study (APCAPS). Int J Epidemiol 2014; **43**(5): 1417-24.		
Data Access: Collaborations welcome, contact Sanjay.Kinra@lshtm.ac.uk		

*CD - Cannot determine

*NR - Not reported

*N/A - Not applicable

Mysore Parthenon Birth Cohort[43]		
Criteria	JW	KH
(CD, NR, NA)*		
1. Was the research question or objective in this paper clearly stated?	Yes	Yes
2. Was the study population clearly specified and defined?	Yes	Yes
3. Was the participation rate of eligible persons at least 50%?	Yes	Yes
4. Were all the subjects selected or recruited from the same or similar populations (including the same time period)? Were inclusion and exclusion criteria for being in the study prespecified and applied uniformly to all participants?	Yes	Yes
5. Was a sample size justification, power description, or variance and effect estimates provided?	cd	CD
6. For the analyses in this paper, were the exposure(s) of interest measured prior to the outcome(s) being measured?	Yes	Yes
7. Was the timeframe sufficient so that one could reasonably expect to see an association between exposure and outcome if it existed?	No – o	No
8. For exposures that can vary in amount or level, did the study examine different levels of the exposure as related to the outcome (e.g., categories of exposure, or exposure measured as continuous variable)?	n/a	Yes
9. Were the exposure measures (independent variables) clearly defined, valid, reliable, and implemented consistently across all study participants?	Yes	Yes
10. Was the exposure(s) assessed more than once over time?	Yes	Yes
11. Were the outcome measures (dependent variables) clearly defined, valid, reliable, and implemented consistently across all study participants?	Yes	Yes
12. Were the outcome assessors blinded to the exposure status of participants?	cd	CD
13. Was loss to follow-up after baseline 20% or less?	Yes	Yes
14. Were key potential confounding variables measured and adjusted statistically for their impact on the relationship between exposure(s) and outcome(s)?	Yes	Yes
Rater #1 initials:	JW	
Rater #2 initials:		KH
Additional Comments (If POOR, please state why):		
Paper		
Krishnaveni GV, Veena SR, Hill JC, Karat SC, Fall CH. Cohort profile: Mysore parthenon birth cohort. Int J Epidemiol 2014: dyu050.		
Data Access: not freely available, collaborators to contact gv.krishnaveni@gmail.com		

*CD – Cannot determine

*NR – Not reported

*N/A – Not applicable

Young Lives[47]		
Criteria	JW	KH
(CD, NR, NA)*		
1. Was the research question or objective in this paper clearly stated?	Yes	Yes
2. Was the study population clearly specified and defined?	Yes	Yes
3. Was the participation rate of eligible persons at least 50%?	Yes	Yes
4. Were all the subjects selected or recruited from the same or similar populations (including the same time period)? Were inclusion and exclusion criteria for being in the study prespecified and applied uniformly to all participants?	Yes	Yes
5. Was a sample size justification, power description, or variance and effect estimates provided?	cd	CD
6. For the analyses in this paper, were the exposure(s) of interest measured prior to the outcome(s) being measured?	Yes	Yes
7. Was the timeframe sufficient so that one could reasonably expect to see an association between exposure and outcome if it existed?	Yes	Yes
8. For exposures that can vary in amount or level, did the study examine different levels of the exposure as related to the outcome (e.g., categories of exposure, or exposure measured as continuous variable)?	n/a	CD
9. Were the exposure measures (independent variables) clearly defined, valid, reliable, and implemented consistently across all study participants?	Yes	Yes
10. Was the exposure(s) assessed more than once over time?	Yes	Yes
11. Were the outcome measures (dependent variables) clearly defined, valid, reliable, and implemented consistently across all study participants?	Yes	Yes
12. Were the outcome assessors blinded to the exposure status of participants?	cd	CD
13. Was loss to follow-up after baseline 20% or less?	Yes	Yes
14. Were key potential confounding variables measured and adjusted statistically for their impact on the relationship between exposure(s) and outcome(s)?	Yes	Yes
Quality Rating (Good, Fair, or Poor) (see guidance)	Good	Good
Rater #1 initials:	JW	
Rater #2 initials:		KH
Additional Comments (If POOR, please state why):		
Paper		
Barnett I, Ariana P, Petrou S, et al. Cohort profile: the Young Lives study. Int J Epidemiol 2013; **42**(3): 701–8.		
Data Access: freely available from http://younglives.qeh.ox.ac.uk		

*CD – Cannot determine

*NR – Not reported

*N/A – Not applicable

Ho Chi Minh City Youth Cohort[53]		
Criteria	JW	KH
(CD, NR, NA)*		
1. Was the research question or objective in this paper clearly stated?	Yes	Yes
2. Was the study population clearly specified and defined?	Yes	Yes
3. Was the participation rate of eligible persons at least 50%?	Yes	Yes
4. Were all the subjects selected or recruited from the same or similar populations (including the same time period)? Were inclusion and exclusion criteria for being in the study prespecified and applied uniformly to all participants?	Yes	Yes
5. Was a sample size justification, power description, or variance and effect estimates provided?	Yes	Yes
6. For the analyses in this paper, were the exposure(s) of interest measured prior to the outcome(s) being measured?	Yes	Yes
7. Was the timeframe sufficient so that one could reasonably expect to see an association between exposure and outcome if it existed?	No	No
8. For exposures that can vary in amount or level, did the study examine different levels of the exposure as related to the outcome (e.g., categories of exposure, or exposure measured as continuous variable)?	n/a	Yes
9. Were the exposure measures (independent variables) clearly defined, valid, reliable, and implemented consistently across all study participants?	Yes	Yes
10. Was the exposure(s) assessed more than once over time?	Yes	Yes
11. Were the outcome measures (dependent variables) clearly defined, valid, reliable, and implemented consistently across all study participants?	Yes	Yes
12. Were the outcome assessors blinded to the exposure status of participants?	cd	CD
13. Was loss to follow-up after baseline 20% or less?	No	No
14. Were key potential confounding variables measured and adjusted statistically for their impact on the relationship between exposure(s) and outcome(s)?	Yes	Yes
Quality Rating (Good, Fair, or Poor) (see guidance)	Good	Good
Rater #1 initials:	JW	
Rater #2 initials:		KH
Additional Comments (If POOR, please state why):		
Paper		
Trang NHHD, Hong TK, Dibley MJ. Cohort profile: Ho Chi Minh City Youth Cohort—changes in diet, physical activity, sedentary behaviour and relationship with overweight/obesity in adolescents. BMJ open 2012; **2**(1): e000362.		
Data Access: Freely available by contacting nguyenhoang_doantrang@yahoo.com (corresponding author) or hongutc@yahoo.com (princple investigator)		

*CD - Cannot determine

*NR - Not reported

*N/A - Not applicable

Cebu Longitudinal Health and Nutrition Survey[44]		
Criteria		
(CD, NR, NA)*	JW	KH
1. Was the research question or objective in this paper clearly stated?	Yes	Yes
2. Was the study population clearly specified and defined?	Yes	Yes
3. Was the participation rate of eligible persons at least 50%?	Yes	Yes
4. Were all the subjects selected or recruited from the same or similar populations (including the same time period)? Were inclusion and exclusion criteria for being in the study prespecified and applied uniformly to all participants?	Yes	Yes
5. Was a sample size justification, power description, or variance and effect estimates provided?	CD	CD
6. For the analyses in this paper, were the exposure(s) of interest measured prior to the outcome(s) being measured?	Yes	Yes
7. Was the timeframe sufficient so that one could reasonably expect to see an association between exposure and outcome if it existed?	Yes	Yes
8. For exposures that can vary in amount or level, did the study examine different levels of the exposure as related to the outcome (e.g., categories of exposure, or exposure measured as continuous variable)?	N/A	Yes
9. Were the exposure measures (independent variables) clearly defined, valid, reliable, and implemented consistently across all study participants?	Yes	Yes
10. Was the exposure(s) assessed more than once over time?	Yes	Yes
11. Were the outcome measures (dependent variables) clearly defined, valid, reliable, and implemented consistently across all study participants?	Yes	Yes
12. Were the outcome assessors blinded to the exposure status of participants?	CD	CD
13. Was loss to follow-up after baseline 20% or less?	No	No
14. Were key potential confounding variables measured and adjusted statistically for their impact on the relationship between exposure(s) and outcome(s)?	Yes	Yes
Quality Rating (Good, Fair, or Poor) (see guidance)	Good	Good
Rater #1 initials:		
Rater #2 initials:	JW	
Additional Comments (If POOR, please state why):		KH
Paper		
Adair LS, Popkin BM, Akin JS, et al. Cohort profile: the Cebu longitudinal health and nutrition survey. Int J Epidemiol 2010: dyq085.		
Data Access: freely available from http://www.cpc.unc.edu/projects/cebu		

*CD – Cannot determine

*NR – Not reported

*N/A – Not applicable

Chinese Metabolic Syndrome Twin Cohort Study[54]		
Criteria	JW	KH
(CD, NR, NA)*		
1. Was the research question or objective in this paper clearly stated?	Yes	Yes
2. Was the study population clearly specified and defined?	No	Yes
3. Was the participation rate of eligible persons at least 50%?	CD	CD
4. Were all the subjects selected or recruited from the same or similar populations (including the same time period)? Were inclusion and exclusion criteria for being in the study prespecified and applied uniformly to all participants?	No	No
5. Was a sample size justification, power description, or variance and effect estimates provided?	cd	No
6. For the analyses in this paper, were the exposure(s) of interest measured prior to the outcome(s) being measured?	Yes	Yes
7. Was the timeframe sufficient so that one could reasonably expect to see an association between exposure and outcome if it existed?	Yes	Yes
8. For exposures that can vary in amount or level, did the study examine different levels of the exposure as related to the outcome (e.g., categories of exposure, or exposure measured as continuous variable)?	N/A	Yes
9. Were the exposure measures (independent variables) clearly defined, valid, reliable, and implemented consistently across all study participants?	Yes	Yes
10. Was the exposure(s) assessed more than once over time?	Yes	Yes
11. Were the outcome measures (dependent variables) clearly defined, valid, reliable, and implemented consistently across all study participants?	Yes	Yes
12. Were the outcome assessors blinded to the exposure status of participants?	CD	CD
13. Was loss to follow-up after baseline 20% or less?	CD	CD
14. Were key potential confounding variables measured and adjusted statistically for their impact on the relationship between exposure(s) and outcome(s)?	No	No
Quality Rating (Good, Fair, or Poor) (see guidance)	Fair/Poor	Fair/Poor
Rater #1 initials:	JW	
Rater #2 initials:		KH
Additional Comments (If POOR, please state why):		
Paper		
Li S, Liu R, Arguelles L, et al. Adiposity trajectory and its associations with plasma adipokine levels in children and adolescents—A prospective cohort study. Obesity 2015.		
Data Access: Not available		

*CD - Cannot determine

*NR - Not reported

*N/A - Not applicable

1982 Pelotas Birth Cohort[55]		
Criteria	JW	KH
(CD, NR, NA)*		
1. Was the research question or objective in this paper clearly stated?	Yes	Yes
2. Was the study population clearly specified and defined?	Yes	Yes
3. Was the participation rate of eligible persons at least 50%?	Yes	Yes
4. Were all the subjects selected or recruited from the same or similar populations (including the same time period)? Were inclusion and exclusion criteria for being in the study prespecified and applied uniformly to all participants?	Yes	Yes
5. Was a sample size justification, power description, or variance and effect estimates provided?	CD	CD
6. For the analyses in this paper, were the exposure(s) of interest measured prior to the outcome(s) being measured?	Yes	Yes
7. Was the timeframe sufficient so that one could reasonably expect to see an association between exposure and outcome if it existed?	Yes	Yes
8. For exposures that can vary in amount or level, did the study examine different levels of the exposure as related to the outcome (e.g., categories of exposure, or exposure measured as continuous variable)?	n/a	Yes
9. Were the exposure measures (independent variables) clearly defined, valid, reliable, and implemented consistently across all study participants?	Yes	Yes
10. Was the exposure(s) assessed more than once over time?	Yes	Yes
11. Were the outcome measures (dependent variables) clearly defined, valid, reliable, and implemented consistently across all study participants?	Yes	Yes
12. Were the outcome assessors blinded to the exposure status of participants?	CD	CD
13. Was loss to follow-up after baseline 20% or less?	No	No
14. Were key potential confounding variables measured and adjusted statistically for their impact on the relationship between exposure(s) and outcome(s)?	Yes	Yes
Quality Rating (Good, Fair, or Poor) (see guidance)	Good	Good
Rater #1 initials:	JW	
Rater #2 initials:		KH
Additional Comments (If POOR, please state why):		
Paper		
Victora CG, Barros FC. Cohort profile: the 1982 Pelotas (Brazil) birth cohort study. Int J Epidemiol 2006; **35**(2): 237-42.		
Data Access: Collaborations welcome http://www. epidemio-ufpel.org.br/projetos_de_pesquisas/estudos/coorte_1982 or email cvictora@terra.com.br.		

*CD - Cannot determine

*NR - Not reported

*N/A - Not applicable

1993 Pelotas Birth Cohort[45,46]		
Criteria	JW	KH
(CD, NR, NA)*		
1. Was the research question or objective in this paper clearly stated?	Yes	Yes
2. Was the study population clearly specified and defined?	Yes	Yes
3. Was the participation rate of eligible persons at least 50%?	Yes	Yes
4. Were all the subjects selected or recruited from the same or similar populations (including the same time period)? Were inclusion and exclusion criteria for being in the study prespecified and applied uniformly to all participants?	Yes	Yes
5. Was a sample size justification, power description, or variance and effect estimates provided?	CD	CD
6. For the analyses in this paper, were the exposure(s) of interest measured prior to the outcome(s) being measured?	Yes	Yes
7. Was the timeframe sufficient so that one could reasonably expect to see an association between exposure and outcome if it existed?	Yes	Yes
8. For exposures that can vary in amount or level, did the study examine different levels of the exposure as related to the outcome (e.g., categories of exposure, or exposure measured as continuous variable)?	N/A	Yes
9. Were the exposure measures (independent variables) clearly defined, valid, reliable, and implemented consistently across all study participants?	Yes	Yes
10. Was the exposure(s) assessed more than once over time?	Yes	Yes
11. Were the outcome measures (dependent variables) clearly defined, valid, reliable, and implemented consistently across all study participants?	Yes	Yes
12. Were the outcome assessors blinded to the exposure status of participants?	CD	CD
13. Was loss to follow-up after baseline 20% or less?	No	No
14. Were key potential confounding variables measured and adjusted statistically for their impact on the relationship between exposure(s) and outcome(s)?	Yes	Yes
Quality Rating (Good, Fair, or Poor) (see guidance)	Good	Good
Rater #1 initials:	JW	
Rater #2 initials:		KH
Additional Comments (If POOR, please state why):		
Paper		
Victora CG, Hallal PC, Araújo CL, Menezes AM, Wells JC, Barros FC. Cohort profile: the 1993 Pelotas (Brazil) birth cohort study. Int J Epidemiol 2008; 37(4): 704-9.		
Gonçalves H, Assunção MC, Wehrmeister FC, et al. Cohort profile update: The 1993 Pelotas (Brazil) birth cohort follow-up visits in adolescence. Int J Epidemiol 2014: dyu077.		
Data Access: Not open access, collaborations welcome http://www.epidemio-ufpel.org.projetos_de_pesquisas/ estudos/coorte_1993		

*CD - Cannot determine

*NR - Not reported

*N/A - Not applicable

Riberao Preto Birth Cohort[56]		
Criteria	JW	KH
(CD, NR, NA)*		
1. Was the research question or objective in this paper clearly stated?	Yes	Yes
2. Was the study population clearly specified and defined?	Yes	Yes
3. Was the participation rate of eligible persons at least 50%?	Yes	Yes
4. Were all the subjects selected or recruited from the same or similar populations (including the same time period)? Were inclusion and exclusion criteria for being in the study prespecified and applied uniformly to all participants	Yes	Yes
5. Was a sample size justification, power description, or variance and effect estimates provided?	CD	CD
6. For the analyses in this paper, were the exposure(s) of interest measured prior to the outcome(s) being measured?	Yes	Yes
7. Was the timeframe sufficient so that one could reasonably expect to see an association between exposure and outcome if it existed?	yes	Yes
8. For exposures that can vary in amount or level, did the study examine different levels of the exposure as related to the outcome (e.g., categories of exposure, or exposure measured as continuous variable)?	N/A	Yes
9. Were the exposure measures (independent variables) clearly defined, valid, reliable, and implemented consistently across all study participants?	Yes	Yes
10. Was the exposure(s) assessed more than once over time?	Yes	Yes
11. Were the outcome measures (dependent variables) clearly defined, valid, reliable, and implemented consistently across all study participants?	Yes	Yes
12. Were the outcome assessors blinded to the exposure status of participants?	CD	CD
13. Was loss to follow-up after baseline 20% or less?	No	No
14. Were key potential confounding variables measured and adjusted statistically for their impact on the relationship between exposure(s) and outcome(s)?	Yes	Yes
Quality Rating (Good, Fair, or Poor) (see guidance)	Good	Good
Rater #1 initials:	JW	
Rater #2 initials:		KH
Additional Comments (If POOR, please state why):		
Paper		
Cardoso VC, Simões V, Barbieri MA, et al. Profile of three Brazilian birth cohort studies in Ribeirão Preto, SP and São Luís, MA. Braz J Med Biol Res 2007; **40**(9): 1165–76.		
Data Access: Not available		

*CD – Cannot determine

*NR – Not reported

*N/A – Not applicable

INCAP Longitudinal Study[57]		
Criteria	JW	KH
(CD, NR, NA)*		
1. Was the research question or objective in this paper clearly stated?	Yes	Yes
2. Was the study population clearly specified and defined?	No	No
3. Was the participation rate of eligible persons at least 50%?	Yes	Yes
4. Were all the subjects selected or recruited from the same or similar populations (including the same time period)? Were inclusion and exclusion criteria for being in the study prespecified and applied uniformly to all participants?	No	No
5. Was a sample size justification, power description, or variance and effect estimates provided?	CD	CD
6. For the analyses in this paper, were the exposure(s) of interest measured prior to the outcome(s) being measured?	No	No
7. Was the timeframe sufficient so that one could reasonably expect to see an association between exposure and outcome if it existed?	Yes	Yes
8. For exposures that can vary in amount or level, did the study examine different levels of the exposure as related to the outcome (e.g., categories of exposure, or exposure measured as continuous variable)?	N/A	CD
9. Were the exposure measures (independent variables) clearly defined, valid, reliable, and implemented consistently across all study participants?	Yes	Yes
10. Was the exposure(s) assessed more than once over time?	Yes	Yes
11. Were the outcome measures (dependent variables) clearly defined, valid, reliable, and implemented consistently across all study participants?	Yes	Yes
12. Were the outcome assessors blinded to the exposure status of participants?	CD	CD
13. Was loss to follow-up after baseline 20% or less?	CD	CD
14. Were key potential confounding variables measured and adjusted statistically for their impact on the relationship between exposure(s) and outcome(s)?	Yes	Yes
Quality Rating (Good, Fair, or Poor) (see guidance)	Fair	Fair
Rater #1 initials:	JW	
Rater #2 initials:		KH
Additional Comments (If POOR, please state why): Fair/Poor - Adolescent follow up of perinatal/ early childhood study of nutrition. Longitudinal anthopological data not available for all participants.		
Paper		
Martorell R. History and Design of the INCAP Longitudinal Study. 1995.		
Data Access: Not available		

*CD - Cannot determine

*NR - Not reported

*N/A - Not applicable

1986 Jamaica Birth Cohort[50]		
Criteria	JW	KH
(CD, NR, NA)*		
1. Was the research question or objective in this paper clearly stated?	Yes	Yes
2. Was the study population clearly specified and defined?	Yes	Yes
3. Was the participation rate of eligible persons at least 50%?	Yes	Yes
4. Were all the subjects selected or recruited from the same or similar populations (including the same time period)? Were inclusion and exclusion criteria for being in the study prespecified and applied uniformly to all participan	Yes	Yes
5. Was a sample size justification, power description, or variance and effect estimates provided?	N/A	CD
6. For the analyses in this paper, were the exposure(s) of interest measured prior to the outcome(s) being measured?	Yes	Yes
7. Was the timeframe sufficient so that one could reasonably expect to see an association between exposure and outcome if it existed?	Yes	Yes
8. For exposures that can vary in amount or level, did the study examine different levels of the exposure as related to the outcome (e.g., categories of exposure, or exposure measured as continuous variable)?	N/A	Yes
9. Were the exposure measures (independent variables) clearly defined, valid, reliable, and implemented consistently across all study participants?	Yes	Yes
10. Was the exposure(s) assessed more than once over time?	Yes	Yes
11. Were the outcome measures (dependent variables) clearly defined, valid, reliable, and implemented consistently across all study participants?	Yes	Yes
12. Were the outcome assessors blinded to the exposure status of participants?	CD	CD
13. Was loss to follow-up after baseline 20% or less?	No	No
14. Were key potential confounding variables measured and adjusted statistically for their impact on the relationship between exposure(s) and outcome(s)?	Yes	Yes
Quality Rating (Good, Fair, or Poor) (see guidance)	Good	Good
Rater #1 initials:	JW	
Rater #2 initials:		KH
Additional Comments (If POOR, please state why):		
Paper		
McCaw-Binns A, Ashley D, Samms-Vaughan M, et al. Cohort profile: the Jamaican 1986 birth cohort study. Int J Epidemiol 2011; 40(6): 1469-76.		
Data Access: Available through Institute of Social and Economic Research Data Bank, University of the West Indies, Mona. Correspoding author affette.mccawbinns@uwimona.edu.jm		

*CD - Cannot determine

*NR - Not reported

*N/A - Not applicable

The Tsimane Amazonian Panel Study (TAPS)[58]		
Criteria	JW	KH
(CD, NR, NA)*		
1. Was the research question or objective in this paper clearly stated?	Yes	Yes
2. Was the study population clearly specified and defined?	Yes	Yes
3. Was the participation rate of eligible persons at least 50%?	No	No
4. Were all the subjects selected or recruited from the same or similar populations (including the same time period)? Were inclusion and exclusion criteria for being in the study prespecified and applied uniformly to all participants?	Yes	Yes
5. Was a sample size justification, power description, or variance and effect estimates provided?	CD	CD
6. For the analyses in this paper, were the exposure(s) of interest measured prior to the outcome(s) being measured?	Yes	Yes
7. Was the timeframe sufficient so that one could reasonably expect to see an association between exposure and outcome if it existed?	CD	CD
8. For exposures that can vary in amount or level, did the study examine different levels of the exposure as related to the outcome (e.g., categories of exposure, or exposure measured as continuous variable)?	N/A	Yes
9. Were the exposure measures (independent variables) clearly defined, valid, reliable, and implemented consistently across all study participants?	Yes	Yes
10. Was the exposure(s) assessed more than once over time?	Yes	Yes
11. Were the outcome measures (dependent variables) clearly defined, valid, reliable, and implemented consistently across all study participants?	Yes	Yes
12. Were the outcome assessors blinded to the exposure status of participants?	CD	CD
13. Was loss to follow-up after baseline 20% or less?	No	No
14. Were key potential confounding variables measured and adjusted statistically for their impact on the relationship between exposure(s) and outcome(s)?	Yes	Yes
Quality Rating (Good, Fair, or Poor) (see guidance)	Good/Fair	Fair
Rater #1 initials:	JW	
Rater #2 initials:		KH
Additional Comments (If POOR, please state why):		
Paper		
Leonard WR, Reyes-García V, Tanner S, et al. The Tsimane'Amazonian Panel Study (TAPS): Nine years (2002-2010) of annual data available to the public. Econ Hum Biol 2015; **19**: 51-61.		
Data Access: freely available. Via http://heller.brandeis.edu/sustainable-international-development/tsimane/index.html		

*CD - Cannot determine

*NR - Not reported

*N/A - Not applicable

Jimma Longitudinal Family Survey of Youth[59]		
Criteria	JW	KH
(CD, NR, NA)*		
1. Was the research question or objective in this paper clearly stated?	Yes	Yes
2. Was the study population clearly specified and defined?	Yes	Yes
3. Was the participation rate of eligible persons at least 50%?	Yes	Yes
4. Were all the subjects selected or recruited from the same or similar populations (including the same time period)? Were inclusion and exclusion criteria for being in the study prespecified and applied uniformly to all participants?	Yes	Yes
5. Was a sample size justification, power description, or variance and effect estimates provided?	CD	Yes
6. For the analyses in this paper, were the exposure(s) of interest measured prior to the outcome(s) being measured?	Yes	Yes
7. Was the timeframe sufficient so that one could reasonably expect to see an association between exposure and outcome if it existed?	Yes	Yes
8. For exposures that can vary in amount or level, did the study examine different levels of the exposure as related to the outcome (e.g., categories of exposure, or exposure measured as continuous variable)?	N/A	Yes
9. Were the exposure measures (independent variables) clearly defined, valid, reliable, and implemented consistently across all study participants?	Yes	Yes
10. Was the exposure(s) assessed more than once over time?	Yes	Yes
11. Were the outcome measures (dependent variables) clearly defined, valid, reliable, and implemented consistently across all study participants?	Yes	Yes
12. Were the outcome assessors blinded to the exposure status of participants?	CD	CD
13. Was loss to follow-up after baseline 20% or less?	No	No
14. Were key potential confounding variables measured and adjusted statistically for their impact on the relationship between exposure(s) and outcome(s)?	Yes	Yes
Quality Rating (Good, Fair, or Poor) (see guidance)	Good	Good
Rater #1 initials:	JW	
Rater #2 initials:		KH
Additional Comments (If POOR, please state why):		
Paper		
Belachew T, Hadley C, Lindstrom D, Getachew Y, Duchateau L, Kolsteren P. Food insecurity and age at menarche among adolescent girls in Jimma Zone Southwest Ethiopia: a longitudinal study. Reprod Biol Endocrinol 2011; **9**(1): 1.		
Data Access: Not freely available, contact authors		

*CD – Cannot determine

*NR – Not reported

*N/A – Not applicable

Kagera Health and Development Survey		
Criteria	Yes	No
(CD, NR, NA)*		
1. Was the research question or objective in this paper clearly stated?	Yes	Yes
2. Was the study population clearly specified and defined?	Yes	Yes
3. Was the participation rate of eligible persons at least 50%?	CD	Yes
4. Were all the subjects selected or recruited from the same or similar populations (including the same time period)? Were inclusion and exclusion criteria for being in the study prespecified and applied uniformly to all partic	Yes	Yes
5. Was a sample size justification, power description, or variance and effect estimates provided?	Yes	CD
6. For the analyses in this paper, were the exposure(s) of interest measured prior to the outcome(s) being measured?	Yes	Yes
7. Was the timeframe sufficient so that one could reasonably expect to see an association between exposure and outcome if it existed?	Yes	Yes
8. For exposures that can vary in amount or level, did the study examine different levels of the exposure as related to the outcome (e.g., categories of exposure, or exposure measured as continuous variable)?	Yes	Yes
9. Were the exposure measures (independent variables) clearly defined, valid, reliable, and implemented consistently across all study participants?	Yes	Yes
10. Was the exposure(s) assessed more than once over time?	Yes	Yes
11. Were the outcome measures (dependent variables) clearly defined, valid, reliable, and implemented consistently across all study participants?	Yes	Yes
12. Were the outcome assessors blinded to the exposure status of participants?	CD	CD
13. Was loss to follow-up after baseline 20% or less?	No	No
14. Were key potential confounding variables measured and adjusted statistically for their impact on the relationship between exposure(s) and outcome(s)?	Yes	Yes
Quality Rating (Good, Fair, or Poor) (see guidance)	Good	Good
Rater #1 initials:	JW	
Rater #2 initials:		KH
Additional Comments (If POOR, please state why):		
Paper		
User's Guide to the Kagera Health and Development Survey Datasets.		
Data Access: freely available. Via http://econ.worldbank.org/WBSITE/EXTERNAL/EXTDEC/EXTRESEARCH/EXTLSMS/0,,contentMDK:21588667~menuPK:4196952~pagePK:64168445~piPK:64168309~theSitePK:3358997,00.html		

*CD - Cannot determine

*NR - Not reported

*N/A - Not applicable

Birth to Twenty		
Criteria	JW	KH
(CD, NR, NA)*		
1. Was the research question or objective in this paper clearly stated?	Yes	Yes
2. Was the study population clearly specified and defined?	Yes	Yes
3. Was the participation rate of eligible persons at least 50%?	Yes	Yes
4. Were all the subjects selected or recruited from the same or similar populations (including the same time period)? Were inclusion and exclusion criteria for being in the study prespecified and applied uniformly to all participants	Yes	Yes
5. Was a sample size justification, power description, or variance and effect estimates provided?	CD	CD
6. For the analyses in this paper, were the exposure(s) of interest measured prior to the outcome(s) being measured?	Yes	Yes
7. Was the timeframe sufficient so that one could reasonably expect to see an association between exposure and outcome if it existed?	Yes	Yes
8. For exposures that can vary in amount or level, did the study examine different levels of the exposure as related to the outcome (e.g., categories of exposure, or exposure measured as continuous variable)?	N/A	Yes
9. Were the exposure measures (independent variables) clearly defined, valid, reliable, and implemented consistently across all study participants?	Yes	Yes
10. Was the exposure(s) assessed more than once over time?	Yes	Yes
11. Were the outcome measures (dependent variables) clearly defined, valid, reliable, and implemented consistently across all study participants?	Yes	Yes
12. Were the outcome assessors blinded to the exposure status of participants?	CD	CD
13. Was loss to follow-up after baseline 20% or less?	No	No
14. Were key potential confounding variables measured and adjusted statistically for their impact on the relationship between exposure(s) and outcome(s)?	Yes	Yes
Quality Rating (Good, Fair, or Poor) (see guidance)	Good	Good
Rater #1 initials:	JW	
Rater #2 initials:		KH
Additional Comments (If POOR, please state why):		
Paper		
Richter L, Norris S, Pettifor J, Yach D, Cameron N. Cohort profile: Mandela's children: the 1990 Birth to Twenty study in South Africa. Int J Epidemiol 2007; **36**(3): 504-11.		
Data Access: Collaborations are established through formal agreements with the principal investigators lrichter@hsrc. ac.za. Data available at http://www.wits. ac.za/birthto20		

*CD - Cannot determine

*NR - Not reported

*N/A - Not applicable

Cape Area Panel Study (CAPS)[49]		
Criteria	JW	KH
(CD, NR, NA)*		
1. Was the research question or objective in this paper clearly stated?	Yes	Yes
2. Was the study population clearly specified and defined?	Yes	Yes
3. Was the participation rate of eligible persons at least 50%?	Yes	Yes
4. Were all the subjects selected or recruited from the same or similar populations (including the same time period)? Were inclusion and exclusion criteria for being in the study prespecified and applied uniformly to all participant	Yes	Yes
5. Was a sample size justification, power description, or variance and effect estimates provided?	Yes	Yes
6. For the analyses in this paper, were the exposure(s) of interest measured prior to the outcome(s) being measured?	Yes	Yes
7. Was the timeframe sufficient so that one could reasonably expect to see an association between exposure and outcome if it existed?	Yes	Yes
8. For exposures that can vary in amount or level, did the study examine different levels of the exposure as related to the outcome (e.g., categories of exposure, or exposure measured as continuous variable)?	N/A	CD
9. Were the exposure measures (independent variables) clearly defined, valid, reliable, and implemented consistently across all study participants?	Yes	Yes
10. Was the exposure(s) assessed more than once over time?	Yes	Yes
11. Were the outcome measures (dependent variables) clearly defined, valid, reliable, and implemented consistently across all study participants?	Yes	Yes
12. Were the outcome assessors blinded to the exposure status of participants?	CD	CD
13. Was loss to follow-up after baseline 20% or less?	No	No
14. Were key potential confounding variables measured and adjusted statistically for their impact on the relationship between exposure(s) and outcome(s)?	Yes	Yes
Quality Rating (Good, Fair, or Poor) (see guidance)	Good	Good
Rater #1 initials:	JW	
Rater #2 initials:		KH
Additional Comments (If POOR, please state why):		
Paper		
Lam D, Ardington C, Branson N, et al. The Cape Area Panel Study: A Very Short Introduction to the Integrated Waves 1-2-3-4-5 (2002-2009) Data. Cape Town: Centre for Social Science Research, University of Cape Town 2011.		
Data Access: Available to download from http://www.caps.uct.ac.za/		

*CD – Cannot determine

*NR – Not reported

*N/A – Not applicable
